# *Mycobacterium tuberculosis* and SARS‐CoV‐2 Co‐Infection: An Efficient Cell Model to Study Lung Host–Pathogen Interactions

**DOI:** 10.1002/cpz1.70433

**Published:** 2026-08-12

**Authors:** Thays Maria Costa de Lucena, Débora Elienai de Oliveira Miranda, Juliana Vieira de Barros Arcoverde, Mariana Souza Bezerra Cavalcanti, Willyene Marilia Dantas, Lindomar José Pena, Michelle Christiane da Silva Rabello, Jaqueline de Azevêdo Silva

**Affiliations:** ^1^ Department of Genetics, Center of Biosciences, Laboratory of Human Genetics and Molecular Biology Federal University of Pernambuco Recife Brazil; ^2^ Molecular Biology Sector, Keizo Asami Institute Federal University of Pernambuco Recife Brazil; ^3^ Laboratory of Virology and Experimental Therapy, Aggeu Magalhães Institute Oswaldo Cruz Foundation Recife Brazil; ^4^ Laboratory of Immunoparasitology, Aggeu Magalhães Institute Oswaldo Cruz Foundation Recife Brazil

**Keywords:** cell model, COVID‐19, host, pathogens, tuberculosis

## Abstract

In less than a year, SARS‐CoV‐2 managed to displace *Mycobacterium tuberculosis* (Mtb) as the leading cause of death worldwide due to a single infectious agent. Both pathogens affect the respiratory tract, mainly the lungs. However, the impact that a possible Mtb + SARS‐CoV‐2 co‐infection can have on the host response is still unknown. Herein we depict a rigorous system to evaluate the complex interaction between two simultaneous infections in vitro in a lung epithelial cell line (A549). Overall, the process includes eight steps: (1) Mtb culture, (2) cell maintenance, (3) preparation of viral stocks, (4) determination of infectious titers, (5) Mtb and SARS‐CoV‐2 co‐infection, (6) determination of intracellular bacterial load, (7) SARS‐CoV‐2 viability test, and (8) optional UV‐C validation assay for infectivity reduction. This comprehensive protocol will allow experimentalists to study the pathogenesis of co‐infection in vitro and facilitate collaborative work in the literature. © 2026 The Author(s). *Current Protocols* published by Wiley Periodicals LLC.

**Basic Protocol**: In vitro coinfection model using Mtb and SARS‐CoV‐2 in human lung epithelial cells

## INTRODUCTION

The co‐infection between tuberculosis (TB), caused by *Mycobacterium tuberculosis* (Mtb), and COVID‐19, caused by the SARS‐CoV‐2 virus, represents a growing challenge to global health, especially in endemic regions (Falzon et al., 2023; Hogan et al., 2020). Mtb remains one of the leading causes of death from a single infectious agent, responsible for ∼1.5 million deaths per year; however, the scenario was quickly reconfigured with SARS‐CoV‐2, whose impact significantly compromised the diagnosis and notification of TB cases, raising concerns about the underreporting of co‐infections and clinical severity, as well as mortality (Migliori et al., 2022; The TB/COVID‐19 Global Study Group, 2022). From a biological perspective, both pathogens are biologically distinct but share tropism for the human respiratory tract, particularly the lungs, where they interact with epithelial and immune cells, modulating crucial pathways of the immune response. Evidence indicates that SARS‐CoV‐2 infection may promote an environment permissive to the progression of Mtb infection and potential reactivation of latent TB infection (LTBI) (Abas et al., 2023; Iovino et al.; 2022).

Despite the clinical and biological relevance of this interaction, the mechanistic understanding of coinfection remains limited, in part due to the constraints of the available experimental models. In vivo models have ethical limitations, high cost, and strict biosafety requirements, while conventional in vitro systems may not adequately reproduce the complexity of the lung microenvironment, but the development of controlled experimental systems becomes essential to analyze the interactions between both pathogens and host (de Lucena et al., 2025; Flores et al., 2022; Rajamanickam et al., 2021).

Thus, this article describes the establishment of an in vitro coinfection model using Mtb and SARS‐CoV‐2 in the human lung epithelial lineage A549, allowing the comparative evaluation of monoinfection and coinfection conditions, enabling the integrated investigation of pathogen viability, host interactions, and activation of cellular and immunological pathways. Compared to previous coinfection models, which often focus on bacterial pathogens such as *Staphylococcus aureus* (Goncheva & Heinrichs, 2023) in the context of COVID‐19, this system allows for an unprecedented assessment of how Mtb can influence SARS‐CoV‐2 infection prior to its onset, as well as modulate the host's cellular response. The successful implementation of this model requires expertise in cell culture and management of highly pathogenic microorganisms, as well as specific training to work in high containment environments. Taken together, this coinfection model offers a robust and reproducible approach to investigate the mechanisms of concomitant pathogenicity, contributing significantly to the advancement of translational research in complex respiratory infections. Although H37Rv and hCoV‐19/Brazil/PE‐FIOCRUZ‐IAM4372/2021 were used in this work, the workflow can be adapted to other validated Mtb and SARS‐CoV‐2 strains. Nevertheless, strain‐specific differences in growth kinetics, aggregation, infectivity, and host‐cell interactions should be considered during protocol optimization.

## IN VITRO COINFECTION MODEL USING Mtb AND SARS‐CoV‐2 IN HUMAN LUNG EPITHELIAL CELLS

This protocol outlines 8 stages: (1) Mtb culture, (2) cell maintenance, (3) preparation of viral stocks, (4) determination of infectious titers, (5) Mtb and SARS‐CoV‐2 co‐infection, (6) determination of intracellular bacterial load, (7) SARS‐CoV‐2 viability test, and (8) optional UV‐C validation assay for infectivity reduction.

*NOTE*: It is imperative to ensure that cell viability and harvest densities remain stable and that the cells utilized in this protocol are free from contamination with mycoplasma. Cell lines should be obtained from authenticated repositories and routinely validated according to institutional quality‐control procedures before experimental use.

*NOTE*: The SARS‐CoV‐2 isolated used in this experiment was hCoV‐19/Brazil/PE‐FIOCRUZ‐IAM4372/2021, deposited in the GISAID database under accession number EPI_ISL_5254314. Viral stocks were propagated, stored, and periodically validated for infectivity and identity according to institutional biosafety and quality‐control procedures.

*NOTE*: The bacteria used in this experiment was Mtb H37Rv (ATCC, cat. no. 27294). Bacterial stocks were obtained from certified collections or validated institutional repositories and periodically assessed for viability, purity, and reproducibility before use.

*NOTE*: For consistency throughout this protocol, the base sterile infection medium will be referred to as SIM (Sterile Infection Medium). Medium containing Mtb will be referred to as Mtb‐IM (Mtb‐Inoculated Medium), and medium containing SARS‐CoV‐2 will be referred to as SV‐IM (SARS‐CoV‐2‐Inoculated Medium).

*CAUTION*: All procedures with Mtb and SARS‐CoV‐2 must be performed in a Biosafety Level 3 (BSL‐3) environment, using standard BSL‐3 practices.

### Materials

7H9 growth medium for Mtb (see recipe)
*Mycobacterium* *tuberculosis* (Mtb), H37Rv (ATCC, cat. no. 27294)
Ogawa‐Kudoh BK (Laborclin, ref. no. 510156)
Maintenance culture medium (see recipe)
Cells:
  Vero (BCRJ Code 0245; Cellosaurus, accession no. CVCL_0059)
  A549 (ATCC CCL‐185; Cellosaurus, accession no. CVCL_0023)
Trypan blue (Gibco, cat. no. 15250061)
Trypsin‐EDTA (Sigma‐Aldrich, cat. no. T4049)
Phosphate‐buffered saline (PBS) (GIBCO, cat. no. 10010023)
Virus and bacteria sterile infection medium (see recipe)
SARS‐CoV‐2, hCoV‐19/Brazil/PE‐FIOCRUZ‐IAM4372/2021 (GISAID database, accession no. EPI_ISL_5254314)
Lysis buffer stock solution (see recipe)
7H10 growth medium for Mtb (see recipe)
Dilution buffer stock solution (see recipe)

Class II and Class III biological safety cabinets (ControlAir Biosafe)
15‐ and 50‐ml conical centrifuge tubes (Thermo Scientific, cat. nos. 339650 and 339653)
Disposable bacteriological inoculation loops
Shaker incubator (e.g., Benchmark INCU‐SHAKER)
Spectrophotometer
Water bath (SolidStell, cat. no. SSDC15L)
10‐, 20‐, 200‐, and 1000‐µl micropipettes (Kasvi) and sterile filtered tips (Fisherbrand or equivalent)
Refrigerated benchtop centrifuge (Eppendorf, cat. no. 05‐400‐61)
Cell counter
Neubauer chamber
Cell‐counting chamber slides with trypan blue stain (Thermo Fisher Scientific, cat. no. C10312)
1.5‐ml microcentrifuge tubes (Thermo Fisher Scientific, cat. no. AM12300)
T25 and T75 cell culture flasks (Thermo Scientific, cat. nos. 156340 and 156472)
CO_2_ incubator (Heraeus HERAcell incubator, Thermo Fisher)
Light microscope (e.g., Nikon Eclipse Ts2‐FL)
5‐, 10‐, and 25‐ml serological pipettes (Fisher Scientific, cat. nos. 501214573, 501214572, and 501214571)
Inverted microscope (e.g., Nikon Eclipse Ts2R)
–80 °C Freezer
2‐ml tubes with screw cap O‐ring (Sarstedt, cat. no. 72.694.006)
6‐, 12‐ and 96‐well cell culture plates (Fisher Scientific, cat. nos. 07‐200‐83, 07‐200‐82, and 951020320)
Vortex mixer
Parafilm (Fisher Scientific, cat. no. NC9595547)
1‐ and 5‐ml sterile syringes (e.g., Fisherbrand, cat. nos. 14‐955‐464 and 14‐955‐458)
Microcentrifuge (Eppendorf, cat. no. 5429000015)
0.22‐µm Corning filter SFCA (Fisher Scientific, cat. no. 09‐754‐13)
Ice container and ice
UV‐C light source

#### Stage 1: *Mycobacterium tuberculosis* culture

This procedure must be performed exclusively in a BSL‐3 laboratory.

##### Day 1: Preparation of bacterial culture

**Figure 1:**
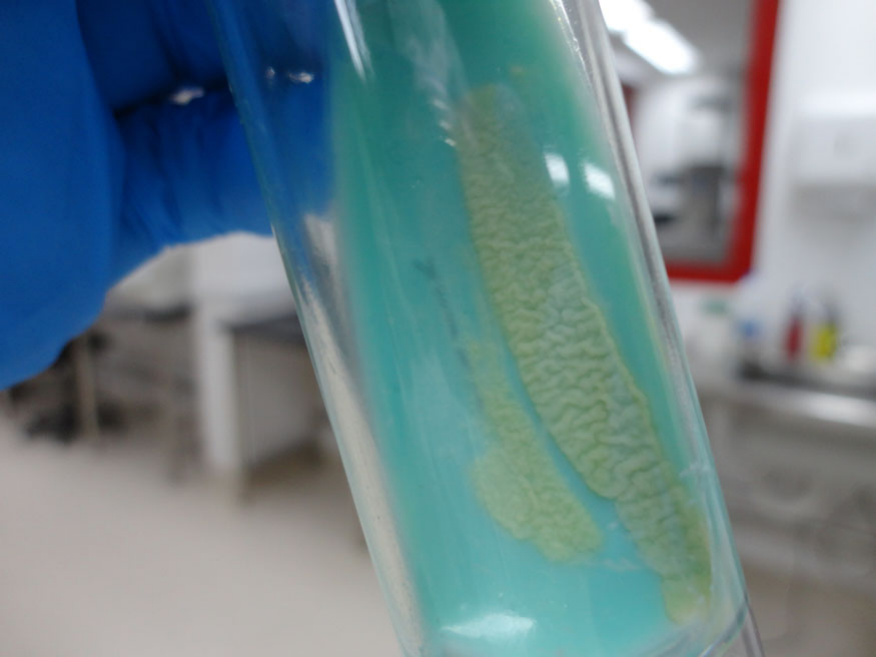
Mtb H37Rv strain in Ogawa‐Kudoh medium.

1 Transfer 5 ml of 7H9 growth medium for Mtb to a 50‐ml conical tube.
2 Using a disposable bacteriological inoculation loop, scrape the Mtb into Ogawa‐Kudoh medium (Fig. 1). Ensuring that there are enough bacteria on the loop, carefully transfer it to the tube with supplemented 7H9 medium, and mix the bacteria into the medium.

3 Place the culture obtained in the previous step in the shaker incubator at 37°C for 4 days.

###### Day 4: Maintenance of bacterial culture

4 Remove the culture from the incubator, and in the biological safety cabinet, add 7 ml of supplemented 7H9 medium to the bacterial culture to replenish nutrients, maintain pH, and reduce cell density, thus achieving efficient growth.
5 Return the culture to the shaker incubator at 37°C.
6 Observe weekly until reaching 28 days of cultivation.
  Mtb is a slow‐growing bacterium, taking ∼28 days to reach the ideal logarithmic phase. If the bacterial culture shows excessive turbidity before 28 days, check the optical density (OD) on the spectrophotometer. More details on how to perform the measurement are in Stage 5, step 62.

#### Stage 2: Cell maintenance (A549 and Vero‐CCL81)

The procedures at this stage can be performed in Biosafety Level 1 (BSL‐1) or Biosafety Level 2 (BSL‐2) laboratories.

##### Day 1: Thawing, replating, and cell counting

7 Pre‐heat the tube containing maintenance culture medium to 37°C in a water bath.
8 Remove the cryovial containing the cells from the liquid nitrogen and thaw them at 37°C in a water bath.
9 In a biosafety cabinet, carefully transfer the cells with a p1000 pipette to a 15‐ml conical tube and add 9 ml of the pre‐warmed maintenance culture medium.
10 Centrifuge the cells 5 min at 200 × *g*, room temperature.
  This process removes any possible freezing residue and forms a pellet.
11 Discard the supernatant.
12 Resuspend cells using a p1000 in 1 ml of pre‐warmed maintenance culture medium, mix gently by pipetting, and transfer to a 15‐ml conical tube.
13 Count viable cells using a cell counter. In a 1.5‐ml tube, mix 10 µl of the cell suspension with 90 µl trypan blue. Add 10 µl of this mixture to one side of a slide inserted into the Newbauer Chamber and count the viable cells in the four quadrants.
14 Ensure the cells have a density of 1 × 10^5^ live cells/cm^2^.
  It is recommended that cells be seeded at a high density to facilitate a more rapid recovery following the thawing process.
15 Transfer the volume of cells for seeding obtained in step 14 to the flask and calculate the difference in maintenance culture medium required.
16 Pipette the pre‐warmed maintenance culture medium into a desired size bottle (T25 bottle should total 5 ml and T75 bottle should total 15 ml).
  A549 cells grow in clusters, so it is recommended to use T25 flasks for their cultivation.
17 Place the flasks with the cells to grow in a CO_2_ incubator (5%) at 37°C until reaching 90% confluence. Check the cultures daily.
  The cells should reach 90% confluence and be prepared for division after 2 to 3 days. Prior to dividing the cells, a visual inspection should be conducted under a light microscope to ascertain whether recovery from freezing has occurred. The cells should be attached to the vial with only a few cells floating around, and their morphology should be unaltered. If cells have not recovered from the thawing process, they often exhibit a more rounded morphology and a reduced degree of connection to neighboring cells. If the cells fail to recover from the freezing process, it is advisable to begin with a fresh vial of cells.

###### Days 3 to 4: Cell division and replication

This section delineates the methodology for two passes, which is imperative to guarantee recuperation from freezing and a stable growth rate prior to initiating subsequent experiments. The process may take up to 10 days from the time of thawing.

18 In a 37°C water bath, pre‐heat the maintenance culture medium, trypsin‐EDTA, and PBS.
19 Aspirate the medium contained in the flasks with a Pasteur pipette or automated serological pipette and wash the cells once with PBS. Aspirate the PBS and add trypsin‐EDTA to cover the monolayer. Incubate for 5 min in an incubator at 37°C.
  For T25 and T75 flasks, add 3 ml and 9 ml of PBS, respectively. For trypsin‐EDTA, add 2 ml for T25 flasks and 6 ml for T75 flasks.
20 Check the detachment of cells under an optical microscope.
  The A549 cell line forms clumps during growth. Under the microscope, it is possible to observe the cells detached from the bottom of the vial, but they are still aggregated. Tap the bottle gently to break up these clumps into single cells.
21 Transfer the cells to a 15‐ or 50‐ml conical tube and stop trypsinization by adding a pre‐warmed maintenance culture medium in the same volume of trypsin‐EDTA as in step 19, considering the size of the flask.
22 Pellet cells by centrifuging 5 min at 200 × *g*, room temperature.
23 Discard the supernatant.
24 Resuspend cells in 1 ml of maintenance culture medium.
25 Count live cells as per step 13.
26 Transfer the required volume of cells and maintenance medium for seeding into the desired flask.
  For 90% confluence in 2 days, seed cells at 4 × 10^4^ live cells/cm^2^. For the same confluence in 3 days, seed at 2.5 × 10^4^ live cells/cm^2^.
27 Place the flask in an incubator at 37°C with 5% CO_2_. Check the cultures daily.
28 Repeat steps 18 through 28 once before using the cells for experiments.

#### Stage 3: Preparation of viral stocks by TCID_50_

##### Day 1: Vero‐CCL81 cell plating

29 In a BSL‐1 or BSL‐2 laboratory, prepare 1 vial of T25 and 1 vial of T75 with Vero‐CCL81 cells at a density of 1 × 10^4^ cells/cm^2^.
30 Place the flasks in an incubator at 37°C with 5% CO_2_ overnight.

###### Days 2 to 5: Inoculation of Vero‐CCL81 cells with SARS‐CoV‐2 strain

Procedures with SARS‐CoV‐2 should be performed in a BSL‐3 laboratory.

31 Transfer the flasks with Vero‐CCL81 cells to the BSL‐3 laboratory.
32 Remove the maintenance culture medium, and then for:
  a. Flask T25 (control), add 400 µl of sterile infection medium and mix carefully with circular movements.
  b. Flask T75 (infection), add 1 ml of the viral strain aliquot and mix carefully with circular movements.
33 Place the flasks in an incubator at 37°C with 5% CO_2_ for 1 hr.
  Every 15 min, observe the bottles and make circular movements to prevent the monolayer from drying out.
34 Using a p1000, add 4.6 ml of sterile infection medium (SIM) to the T25 bottle and 14 ml SIM to the T75 bottle.
35 Store the vials in the incubator at 37°C with 5% CO_2_ for 72 hr.
36 Remove from the incubator and store the bottles in the freezer at –80°C for 24 hr.

###### Day 6: Virus harvesting

**Figure 2:**
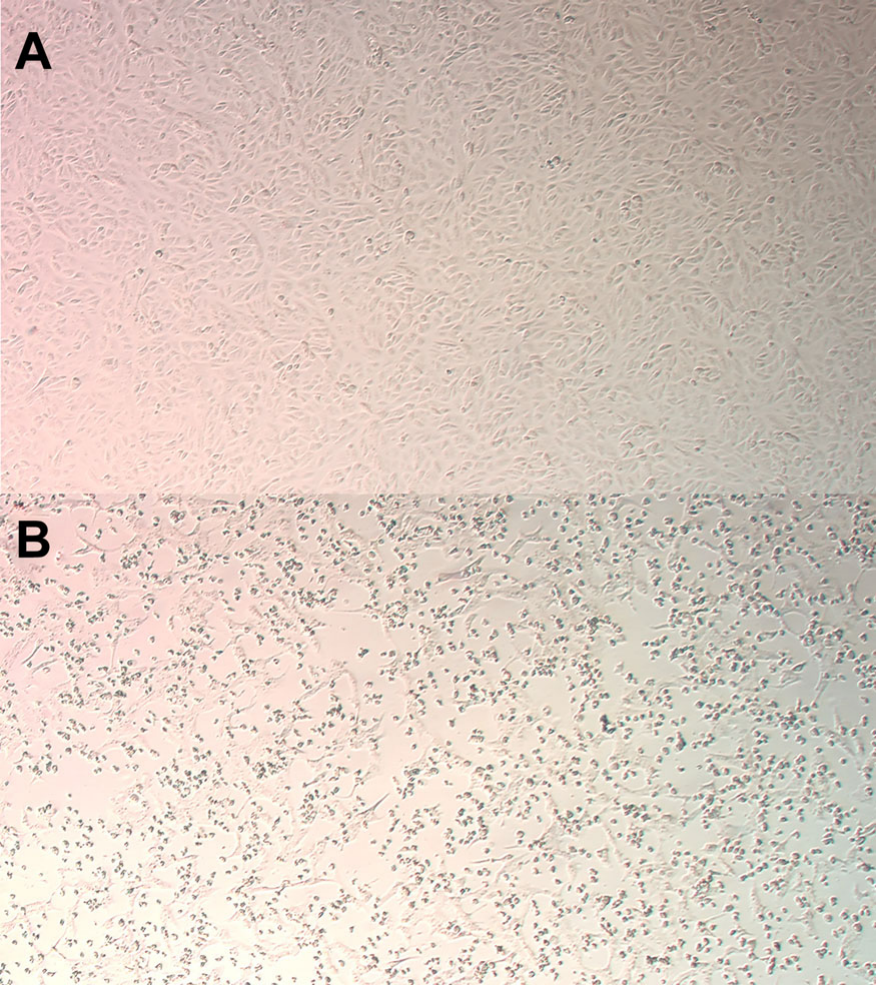
Vero CCL‐81 cells (control and SARS‐CoV‐2 infected). (**A**) Uninfected cells used as controls. (**B**) SARS‐CoV‐2 infected cells.

37 Thaw the flasks at room temperature.
38 View the flasks under an optical microscope. Check whether there is contamination in the T25 and whether the cytophatic effect (CPE) is visible in vial T75 (Fig. 2).

39 Discard the T25 flask.
40 Transfer the contents of the T75 flask to a 50‐ml conical tube.
41 Remove cellular debris by centrifugation for 10 min at 3500 × *g*, 4°C.
42 Transfer the supernatant to a new 50‐ml conical tube and discard the tube containing the pellet.
43 Identify tubes with screw caps and sealing rings and make aliquots of 150 µl each to be used in the next step.

#### Stage 4: Determination of SARS‐CoV‐2 infectious titers by TCID_50_ assay

##### Day 1: Plating of Vero‐CCL81 cells for infection

**Figure 3:**
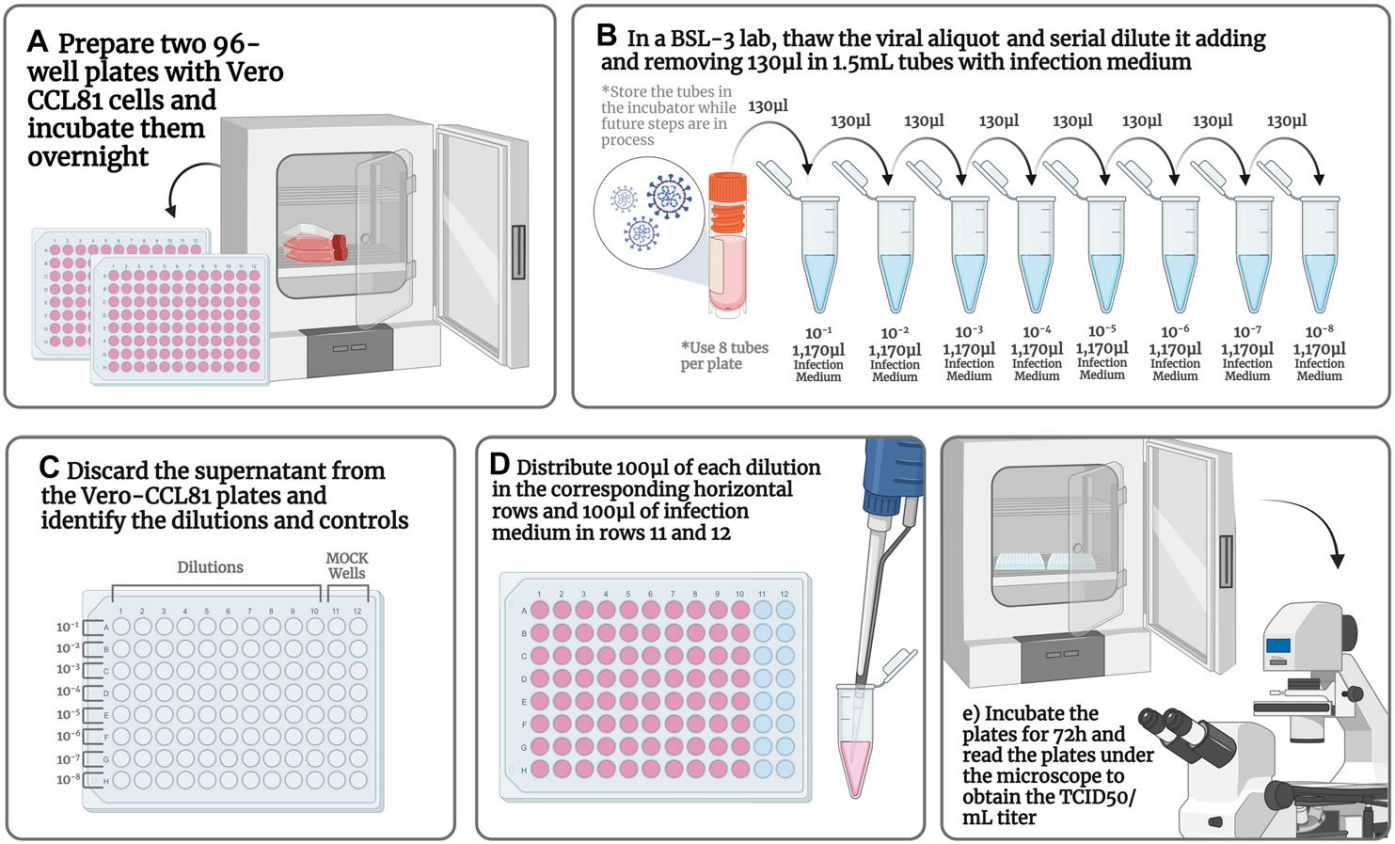
Workflow for TCID_50_ assay. (**A**) Vero‐CCL81 incubation. (**B**) Dilution of the viral aliquot. (**C**) Identification of dilutions and MOCK in the 96‐well plate. (**D**) Insert the dilutions into the corresponding wells. (**E**) Incubate the plates.

44 Prepare Vero‐CCL81 cells in two 96‐well plates at 1 × 10^4^ cells/well (Fig. 3A).
  The preparation of two plates is to demonstrate experimental replication, ensuring experimental replication, consistency of results in different replicates, reduction of experimental errors, e.g., contamination and inadequate handling, and robust statistical analysis.

45 Place the plates in a 37°C incubator with 5% CO_2_ overnight.
  After the incubation period, check under the microscope whether the cells have formed a compact confluent monolayer in each well.
  This stage can be performed in a BSL‐1 or BSL‐2 laboratory. The remaining stages must be performed under BSL‐3 containment.

###### Day 2: TCID_50_ assay

This procedure with infectious SARS‐CoV‐2 must be performed in a BSL‐3 laboratory.

46 Transfer the plates to the BSL‐3 laboratory and place them in a 37°C incubator with 5% CO_2_ until virus inoculation.
47 Thaw a viral aliquot at room temperature.
48 Prepare 10× serial virus dilutions as follows:
  a. Distribute 8 tubes (1.5‐ml) in two rows (totaling 16 tubes, 1 row for each plate).
  b. Identify the tubes according to the dilution (10^−1^, 10^−2^, 10^−3^, 10^−4^, 10^−5^, 10^−6^, 10^−7^, 10^−8^).
  c. Using a p1000, add 1170.0 µl of SIM to all tubes.
  d. Vortex the viral aliquot
  e. Add 130 µl of the virus to the tubes labeled 10^−1^, mix thoroughly with the pipette and vortex briefly.
  f. For subsequent dilutions, remove 130 µl of the newly created dilution and insert it into the next tube, as shown in Figure 3B.
    For each newly created dilution, vortex briefly. This ensures that the contents of the tubes are uniform and there is no difference in the plate reading.
  g. Store these tubes with the respective dilutions in the incubator at 37°C with 5% CO_2_.
49 Discard supernatants from plates containing Vero‐CCL81 cells.
50 Identify the dilutions and controls on the plates, as shown in Figure 3C.
51 Distribute 100 µl SIM in rows 11 and 12, which correspond to “mock”.
52 Distribute 100 µl of each dilution in the corresponding horizontal rows (Fig. 3D).
53 Store the plates in the incubator at 37°C with 5% CO_2_ for 72 hr.

###### Day 5: Reading of plates by optical microscopy and calculation of viral titer

54 Remove the plate from the incubator and read the wells under an optical microscope (Fig. 3E).
55 Start reading the “mock” wells and check if there is no contamination.
56 In infected wells, one must look carefully, including in peripheral regions, to see if there is a CPE.
  If a particular well raises doubts as to whether or not the CPE is present, view it and compare it again with the mock wells.
  Use a 96‐well plate reference sheet to help identify wells that do or do not show CPE. If a CPE is present in a well, mark a “+” on the reference sheet. If there is no CPE in a well, mark a “–” on the reference sheet.
57 Repeat steps 45 through 47 for the second board.
58 After reading both plates, obtain the TCID_50_/ml titer for this stock.
  Both plates must show the same viral titer or a deviation ≤5%.

#### Stage 5: Co‐infection of the A549 cell line by Mtb and SARS‐CoV‐2

To carry out this step, all previous steps must have been carried out.

##### Day 1: Plating of A549 cell line

59 In a BSL‐1 or BSL‐2 laboratory, seed A549 cells in a 12‐well plate at a concentration of 5 × 10^4^ cells/well, considering 1 ml of maintenance culture medium per well.
60 To obtain ideal confluence, keep the plates in an incubator at 37°C with CO_2_ for 48 hr.

###### Day 2: Preparation of bacterial inoculum and infection of A549 cells

Mtb handling must be carried out exclusively in BSL‐3 laboratories.

61 Transfer the plates to the BSL‐3 laboratory and store them in the incubator at 37°C with 5% CO_2_ until infection is performed.
62 After 28 days of bacterial growth, check the OD using the spectrophotometer:
  a. Pipette 2 ml of supplemented 7H9 medium into a cuvette to serve as the “blank”.
  b. Pipette 2 ml of the bacterial culture into another cuvette.
    Mtb is a pathogen whose main means of transmission is the emission of aerosol particles, therefore, when leaving the biosafety cabinet and taking the sample to the spectrophotometer, place Parafilm in the upper region of the cuvette containing the bacterial culture.
  c. Set the spectrophotometer to measure at 600 nm.
  d. An OD at 600 nm (OD600) of 0.6 represents ∼1 × 10^8^ bacteria/ml may be used as a preliminary estimate of bacterial density. However, laboratories are encouraged to establish strain‐specific correlations between OD600 and CFU counts for inoculum standardization.
    Optical density measurements provide an estimate of total bacterial biomass and should not replace viability determination by CFU enumeration whenever accurate bacterial quantification is required.
63 To obtain an appropriate volume of bacteria for ∼1 × 10^8^ bacteria in 5 ml of supplemented 7H9 medium, transfer 1 ml of the culture of bacteria to a 15‐ml conical tube and add 4 ml of supplemented 7H9.
64 Centrifuge 10 min at 1800 × *g*, 37°C.
65 Discard the supernatant.
66 Resuspend the bacterial pellet in 1 ml SIM in the 15‐ml conical tube to obtain Mtb‐IM.
67 Place the tube on a 15‐ml conical tube rack to provide support, and with the 1‐ml syringe, resuspend the bacterial solution 10 times or until the bacteria are completely disaggregated.
  Mycobacteria grow in clusters, which makes it difficult to break them up. If the volume of 1 ml is too low and makes it difficult for the syringe to reach the culture, add more medium, but do not exceed 10 ml.
68 Add 9 ml SIM at a tenfold dilution (homogenize with a pipette) to ∼1 × 10^7^ cells/ml.
69 Store the culture at 37°C until infection is performed.
70 Remove the culture plates from the incubator and remove the supernatant.
71 Add a multiplicity of infection (MOI) of 10:1 (bacteria:cell) to the wells specified for bacterial mono‐infection and co‐infection with Mtb‐IM, corresponding to 1 ml per well.
  For infection of A549 cells in a 12‐well plate, consider the cell population of each well as 2 × 10^5^, adjust the concentration of bacteria to 2 × 10^6^_,_ and calculate the required volume to 1 ml per well.
72 Incubate the plates for 2 hr at 37°C with 5% CO_2_.
73 Carefully remove the supernatant from the wells and avoid disturbing the cell's monolayer.
74 Wash the cells twice with 0.5 ml PBS.
75 Add 1 ml SIM.
76 Incubate the plates for 1 hr at 37°C with 5% CO_2_.
77 Repeat steps 73 and 74.
78 Add 1 ml SIM and store the plates in the incubator at 37°C with 5% CO_2_ for 24 hr.

###### Day 3: Preparation of viral inoculum and infection of A549 cells

79 Continue assuming a cell population of 2 × 10^5^ cells/well. Dilute the viral stock (obtained in stage 3) to an MOI of 1:1 (virus:cell) in a volume of 100 µl per well with SIM to obtain SV‐IM.
80 Remove the plates from the incubator, and in the biosafety cabinet, remove the medium from the plates.
81 Wash twice with PBS at ∼0.5 ml per well.
82 Remove the PBS and add 100 µl of the SV‐IM to the wells corresponding to SARS‐CoV‐2 monoinfection and co‐infection.
  It is important to have a “mock” well, where only 100 µl of infection medium but no virus will be added.
83 Return the plate to the incubator at 37°C with 5% CO_2_ for 1 hr.
  Every 10 min, gently make circular movements on the plates. This procedure serves to redistribute the inoculum and prevent the cell monolayer from drying out.
84 Return the plates to the biosafety cabinet and remove the viral inoculum.
85 Wash the wells twice with 0.5 ml PBS and add 1 ml SIM to each well.
86 Incubate the plates at 37°C with 5% CO_2_ for 72 hr.

###### Day 5: Infection harvesting

87 Remove the plates from the incubator.
88 In the biosafety cabinet, collect the infection medium with a p1000, transfer to properly identified 1.5‐ml tubes, and set aside the culture plates.
  After collecting the supernatants, do not discard the plate. It should be reserved to continue the experiments, as there will still be viable cells at the bottom of the plate well that can be used for RNA extraction and determination of the intracellular bacterial load.
89 Microcentrifuge the tubes 1 min at 15,000 × *g*, room temperature.
90 Using a 1‐ml syringe, remove the supernatants from the tubes without disturbing the pellet.
  The tubes containing Mtb mono‐infection and co‐infection samples should be stored. These cell products will be returned to their respective culture wells for Mtb intracellular bacterial load testing and RNA extraction. Further details are described in Stage 6.
91 Attach the syringe to a 0.22‐µm filter and filter the supernatant into a new tube.
  Supernatants from SARS‐CoV‐2 mono‐infection and co‐infection wells can be used for quantification of viral particles by TCID_50_ and protein analysis by flow cytometry. To do this, it is necessary to aliquot these supernatants into different appropriately labeled tubes. The samples obtained can be measured immediately or stored at –80°C until needed.
92 Store all supernatants at –80°C.
93 Return the pellet to its respective well and perform RNA extraction using the Trizol method or proceed to the determination of intracellular bacterial load (Stage 6, steps 94 to 101).

#### Stage 6: Determination of intracellular bacterial load

**Figure 4:**
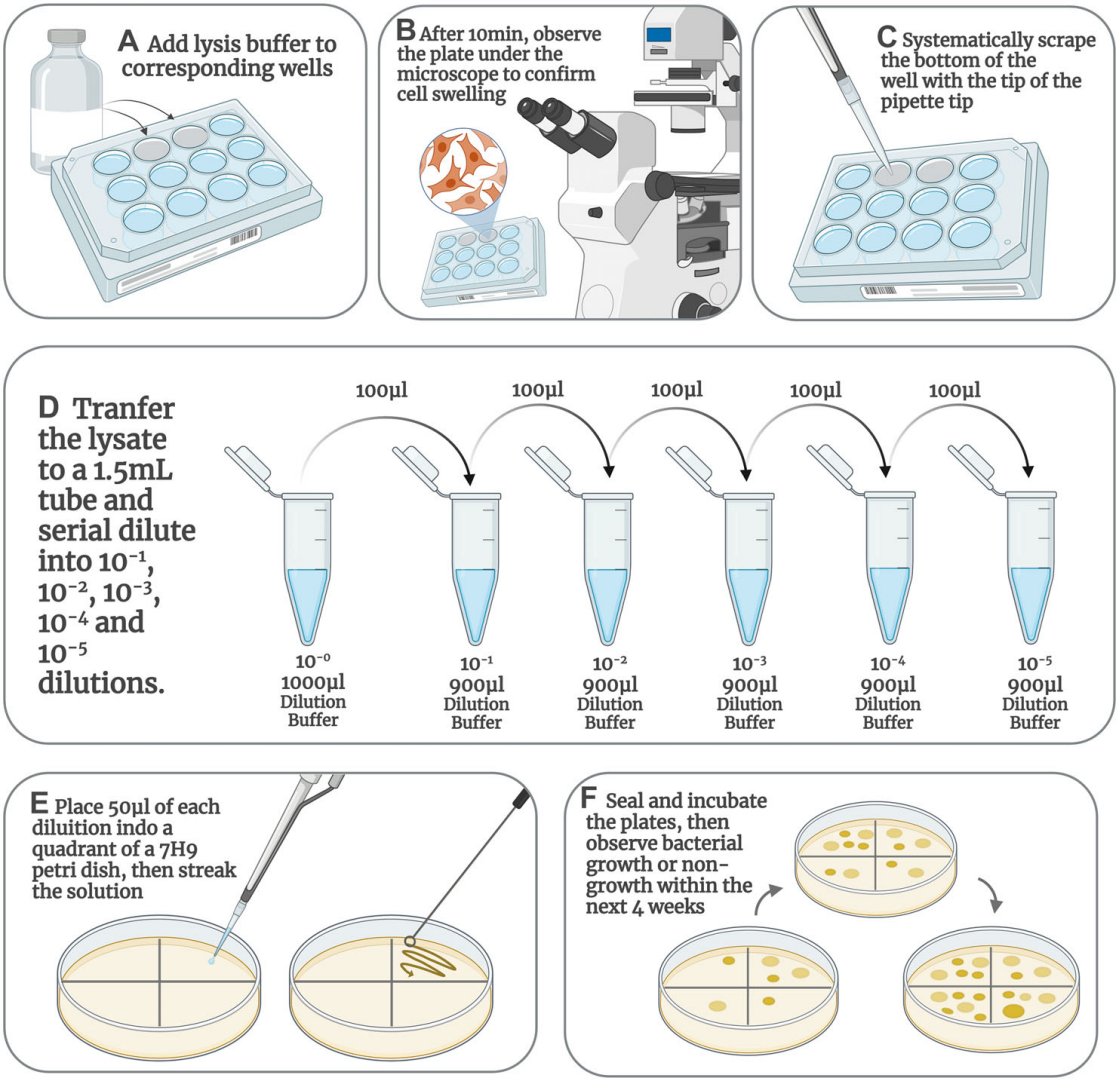
Workflow for determination of intracellular bacterial load. (**A**) Lysis buffer in the specific well. (**B**) Observing the activity of the lysis buffer under the microscope. (**C**) Technical scraping to obtain better lysates. (**D**) Serial dilution of lysates. (**E**) Plating of dilutions. (**F**) CFUs count.

94 In the reserved plate, return the pellets obtained after centrifugation to the respective wells (step 90).
95 Using a p1000, add 1 ml of lysis buffer to the wells corresponding to Mtb mono‐infection and co‐infection (Fig. 4A).

**Figure 5:**
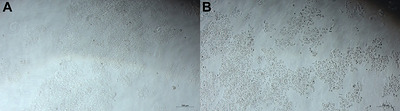
Mtb infection and wells with lysis buffer. (**A**) A549 + Mtb and with lysis buffer. (**B**) A549 + Mtb + SARS‐CoV‐2 and with a lysis buffer.

96 Incubate the plate at room temperature for 10 min to promote cell lysis (Fig. 4B).
97 Observe the cells under an optical microscope; you will be able to see the Mtb‐infected cells “swollen” (Fig. 5).
  Note the difference between uninfected cells and cells with lysis buffer.

98 Using a p1000, systematically scrape the well with a pipette tip (Fig. 4C), and pipette the suspension equally into all wells up and down. This technique will promote complete and linear lysis of the cells.
99 In 1.5‐ml tubes, serially dilute the lysed cells in suspension by 10^−1^, 10^−2^, 10^−3^, 10^−4^, and 10^−5^ using the dilution buffer. Follow the image in Figure 4D to perform the dilutions.
  Save all unplated tubes, diluted and undiluted, until the end of the colony forming unit (CFU) count.
100 Place 50 µl of each dilution on a quadrant of the appropriate 7H10 agar plate. Spread the lysed cell solution onto the sterile 7H10 plate with a disposable inoculating loop and allow it to dry completely (Fig. 4E).
101 Seal the plates with Parafilm and incubate at 37°C. Colony forming units (CFUs) will begin to appear 2 to 3 weeks after inoculation (Fig. 4F).
  Perform a 7H10 control plate with the reagents used in the bacterial culture to ensure the absence of contamination.
  It is important that the plates are checked after 48 hr of inoculation to ensure the absence of contamination.
  When colonies begin to appear, they should be counted weekly to check whether there has been more growth in CFUs from one week to the next. Plates with 7H10 should be stored for up to 28 days. After this period, they should be discarded.

#### Stage 7: Viability test of SARS‐CoV‐2 by TCID_50_ assay

##### Day 1: Plating of Vero‐CCL81 cells for infection

**Figure 6:**
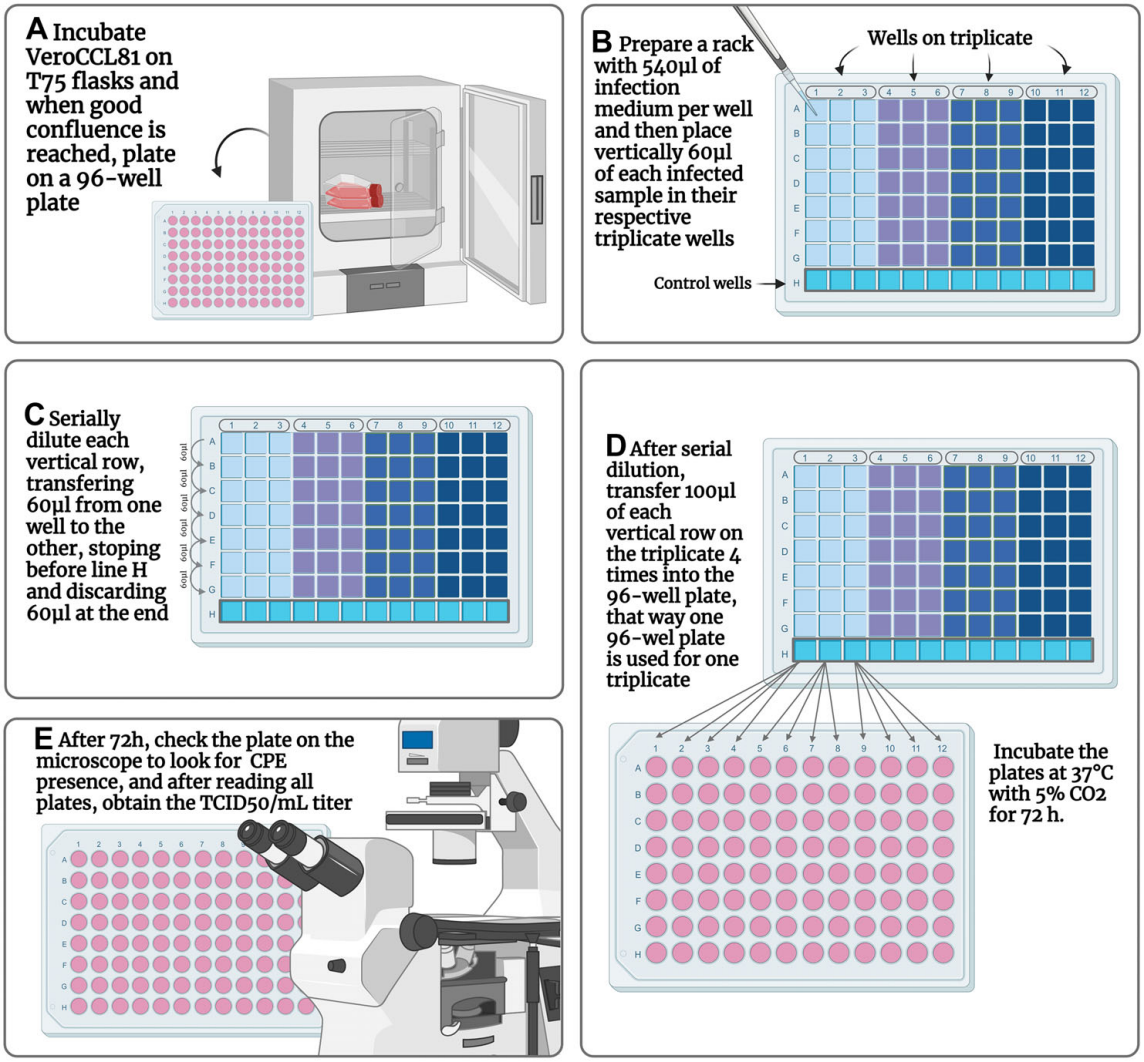
Workflow of SARS‐CoV‐2 viability test. (**A**) Vero‐CCL81 incubation. (**B**) Preparation of the rack with the supernatants and infection medium. (**C**) Serial dilution in the rack. (**D**) Every third vertical row of the rack, transfer to a 96‐well plate. (**E**) Observation of CPE under the microscope.

102 Plating of Vero‐CCL81 cells (Fig. 6A) for this step can be performed as per Stage 4, step 35 and 36.
  Calculate the number of possible 96‐well plates according to the number of samples that need to be quantified. Each triplicate of samples is equivalent to a 96‐well plate. Each rack is equivalent to 4 triplicate samples (Fig. 6B), resulting in a total of four 96‐well plates.

###### Day 2: Rack preparation and infection of Vero‐CCL81 cells with supernatants

103 Forward the 96‐well plates to the BSL‐3 laboratory.
104 In a biosafety cabinet, distribute 540 µl SIM into each well of the rack (Fig. 6B).
105 Add 60 µl of each sample to the first well of each vertical row.
106 Perform serial dilution of each vertical row, transferring 60 µl from one well to the other (Fig. 6C).
  In well G of each vertical row, add 60 µl and then discard the sample. Do not pipette anything into well H of each vertical row because it is the control.
107 Transfer 100 µl from the rack to the 96‐well plate (Fig. 6D).
  Each vertical row will be added 4× in the 96‐well plate (also vertically), where each triplicate (obtained from cell culture) of samples will be a 96‐well plate.
108 Incubate the plates at 37°C with 5% CO_2_ for 72 hr.

###### Day 5: Reading of plates by optical microscopy and calculation of viral titer

**Figure 7:**
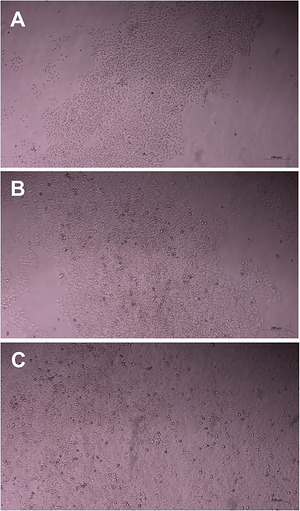
TCID50 assay and CPE visualization. (**A**) Control well (A549 only). (**B**) A549 + Mtb + SARS‐CoV‐2. (**C**) A549 + SARS‐CoV‐2.

109 Perform the procedure as per Stage 4, steps 54 to 58, and both can be viewed as per Figure 7.

#### Stage 8 (optional): UV‐C exposure validation assay

##### Day 1: preparation of Vero‐CCL81 cells

This process can be performed in BSL‐1 or BSL‐2.

**Figure 8:**
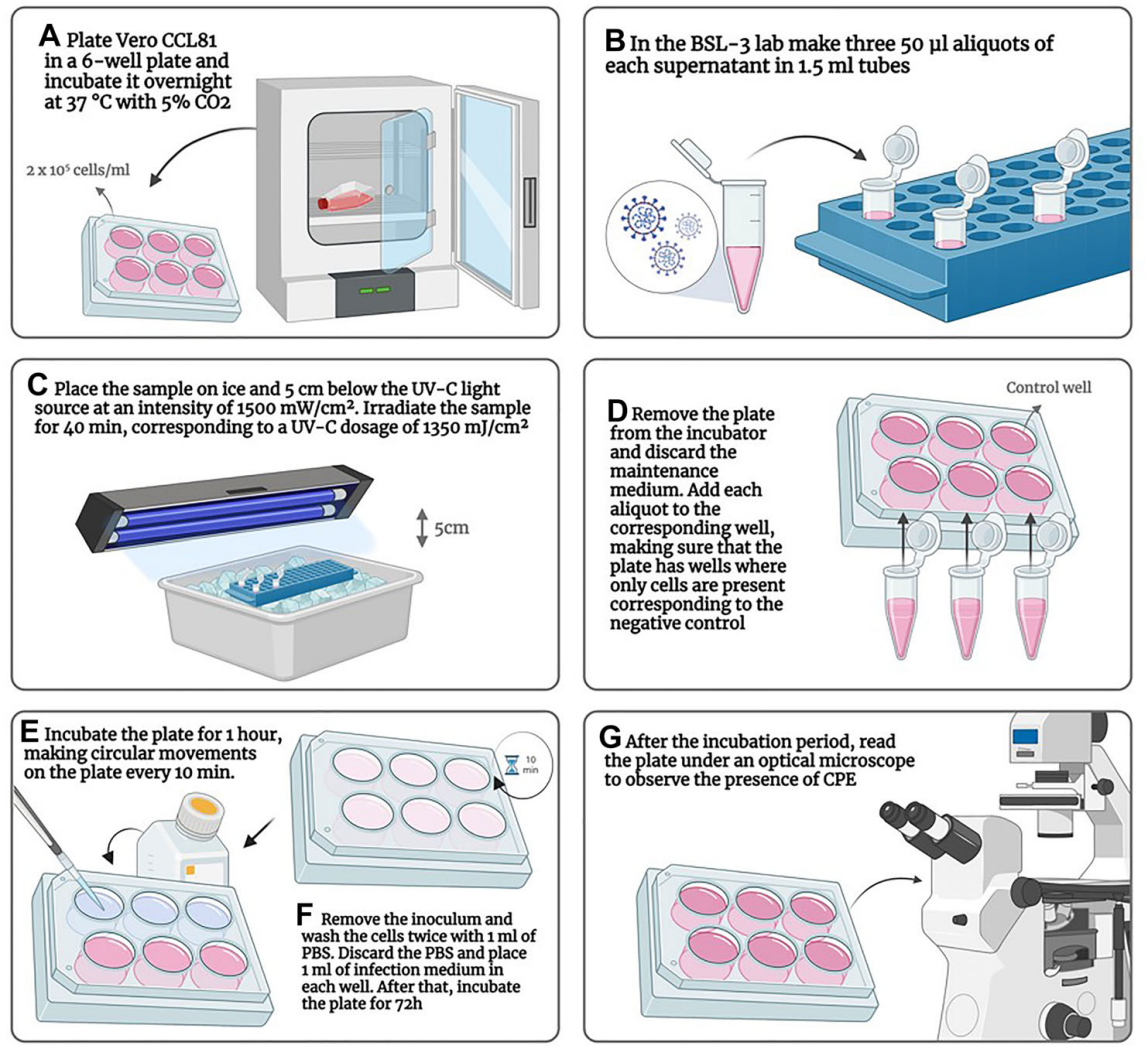
Workflow decontamination supernatant. (**A**) Vero CCL‐81 incubation. (**B**) Aliquots of the supernatant. (**C**) UV‐C radiation at 5 cm for 40 min. (**D**) Preparation of the 6‐well plate with the viral aliquots and negative control wells (cells only). (**E**) Incubation of the aliquots for 1 hr and circular movements every 10 min. (**F**) Washing the wells and adding infection medium. (**G**) Incubation for 72 hr and observation of CPE under the microscope.

110 In a 6‐well culture plate, plate a concentration of Vero CCL‐81 cells corresponding to 2 × 10^5^ cells/ml in each well (Fig. 8A).

111 Incubate the plate overnight in an incubator at 37°C with 5% CO_2_.

###### Day 2: Sample decontamination test

This process must be performed in BSL‐3.

112 After the incubation period, transfer the plate to the BSL‐3.
113 Make 3 aliquots of 50 µl of each supernatant in 1.5‐ml tubes (Fig. 8B).
  The 3 aliquots must come from the same supernatant, and each one must be inoculated into three different wells.
114 Place the sample on ice and 5 cm below the UV‐C light source at an intensity of 1500 mW/cm² as per Figure 8C.
  Tubes must remain open during UV‐C exposure because most laboratory plastics partially block UV‐C transmission.
115 Irradiate the sample for 40 min, corresponding to a UV‐C dosage of 1350 mJ/cm².
  It is necessary to check how long it takes for the UV‐C light source used to reach this intensity and dosage, and then count the 40 min.
116 Remove the plate from the incubator and discard the maintenance medium.
117 Add each aliquot to the corresponding well for the decontamination test (Fig. 8D).
  Make sure that the plate has wells corresponding to the negative control where there will only be cells without insertion of the sample that will be decontaminated.
118 Incubate in the incubator for 1 hr, and every 10 min make circular movements on the plate (Fig. 8E).
119 Remove the inoculum and wash the cells twice with 1 ml PBS (Fig. 8F).
120 Discard the PBS and place 1 ml SIM in each well. Incubate the plate in an incubator at 37°C with 5% CO_2_ for 72 hr.

###### Day 5: Reading the plate by optical microscopy

121 After the incubation period, read the plate under an optical microscope to observe the CPE (Fig. 8G).
  The absence of CPE in the wells corresponding to viral aliquots suggests a reduction in infectivity under the specific experimental conditions tested.

## REAGENTS AND SOLUTIONS

### 7H9 growth medium for Mtb

Middlebrook 7H9 medium is a liquid culture medium specific for mycobacteria. It can be prepared in BSL‐1 or BSL‐2 laboratories. Weigh 2.35 g of Middlebrook 7H9 medium (BD DIFCO, cat. no. DF0713‐17‐9) and place it in a sterilized 500‐ml bottle. Add 449 ml of ultrapure water and 1 ml of UltraPure glycerol (Thermo Fisher Scientific, cat. no. 15514011). Perform circular movements to homogenize. Autoclave at 121°C for 10 min.

To continue with the supplementation, the medium must be at room temperature. Send the bottle to the BSL‐3 laboratory and distribute 45 ml 7H9 medium to 50‐ml conical tubes in the biosafety cabinet. Add 10% oleic acid‐albumin‐dextrose‐catalase (OADC) stock solution (BD BBL, cat. no. 211886) (5 ml in a final volume of 50 ml) and 0.05% Tween 80 (Thermo Fisher Scientific, cat. no. 28329). Mix the contents by vortexing the tube for 10 s or until the Tween 80 is completely dissolved. Store up to 6 weeks at 4°C. For long‐term preservation, Mtb stocks may be prepared in supplemented 7H9 containing glycerol as cryoprotectant and stored at or below –70°C. Frozen stocks should be periodically validated by CFU determination to ensure viability and reproducibility.

### 7H10 growth medium for Mtb

Middlebrook 7H10 medium is a solid culture medium used for the cultivation of mycobacteria. Prepare the 7H10 medium in a BSL‐2 laboratory. Weigh 4.75 g of Middlebrook 7H10 medium (BD DIFCO, cat. no. 11799042) and transfer the contents to a 500‐ml Erlenmeyer flask (e.g., Fisher Scientific, cat. no. FB 501500). Add 223.75 ml of ultrapure water and 1.25 ml of UltraPure glycerol (Thermo Fisher Scientific, cat. no. 15514011), microwave for 3 min, and every 1 min remove and perform circular movements to dissolve the contents more efficiently. Autoclave at 121°C for 10 min.

After sterilization, this medium must be supplemented. Wait for it to cool to room temperature and use a thermometer to check the temperature of the medium, still in a liquid state. When it reaches 50°C, add OADC stock solution (BD BBL, cat. no. 211886) to the Erlenmeyer flask to obtain a final concentration of 10% and quickly and safely plate by inserting 25 ml of supplemented medium into each disposable Petri dish (Thermo Scientific, cat. no. 150350). Allow the medium to dry completely and seal the plates with Parafilm (Fisher Scientific, cat. no. NC9595547). Place a control plate in the incubator at 37°C for 24 hr to check for possible contamination. If there is no contaminant growth, transport the plates to the BSL‐3 laboratory and store up to 2 weeks at 4°C.

### Dilution buffer stock solution

Prepare dilution buffer in a BSL‐2 laboratory. In a 250‐ml flask, add Tween 80 (Thermo Fisher Scientific, cat. no. 28329) until a final concentration of 0.05% is reached in 250 ml of phosphate‐buffered saline (PBS) (GIBCO, cat. no. 10010023). Autoclave the solution at 121°C for 10 min, allow it to cool to room temperature, and filter the entire volume through a 0.22‐µm filter. Transport to BSL‐3 and store up to 6 weeks at room temperature.

### Lysis buffer stock solution

Prepare lysis buffer in a BSL‐2 laboratory. In a 250‐ml flask, add Tween 80 (Thermo Fisher Scientific, cat. no. 28329) until a final concentration of 0.1% is reached in 250 ml of deionized water. Autoclave the solution at 121°C for 10 min, allow it to cool to room temperature, and filter the entire volume through a 0.22‐µm filter. Transport to BSL‐3 and store up to 6 weeks at room temperature.

### Maintenance culture medium

The maintenance culture medium contains components that are necessary for cell proliferation and culture adaptation after thawing and passages. It can be prepared in a BSL‐1 or BSL‐2 laboratory in a biosafety cabinet. To a 50‐ml conical tube, add 1% penicillin‐streptomycin (500 µl of 100×; Gibco, cat. no. 15140122; final concentration 100 IU penicillin, 100 µg/ml streptomycin), 10% heat‐inactivated fetal bovine serum (FBS) (5 ml; Gibco, cat. no. 12657029), and 44.5 ml Dulbecco's modified Eagle medium (DMEM) containing 4.5 g/L d‐glucose, with 4 mM l‐glutamine and 1 mM sodium pyruvate (Capricorn Scientific, cat. no. DMEM‐HPA). Mix the tube well with circular movements or by inversion. Store up to 6 weeks at 4°C.

### Virus and bacteria sterile infection medium

The infection medium is adapted to promote infection of cells, specifically viruses and bacteria. Prepare the viral and bacterial infection medium in a BSL‐1 or BSL‐2 laboratory. To prepare an infection medium, 2% FBS is required. In a 50‐ml conical tube, add heat‐inactivated FBS (Gibco, cat. no. 12657029) to obtain a final concentration of 2% and penicillin‐streptomycin (Gibco, cat. no. 15140122) to obtain a final concentration of 1%, then add 48.5 ml DMEM (Capricorn Scientific, cat. no. DMEM‐HPA). Shake the bottle well by rotating or inverting it. Mix the bottle thoroughly by swirling or inverting. Transport to the BSL‐3 laboratory and store up to 6 weeks at 4°C. For consistency throughout this protocol, the base sterile infection medium will be referred to as SIM (Sterile Infection Medium). Medium containing Mtb will be referred to as Mtb‐IM (Mtb‐Inoculated Medium), and medium containing SARS‐CoV‐2 will be referred to as SV‐IM (SARS‐CoV‐2‐Inoculated Medium).

## COMMENTARY

### Limitations and Troubleshooting

Our experimental project was to co‐infect a lung epithelial tissue cell line with Mtb and SARS‐CoV‐2 and obtain samples to verify the immune response pathways that may be activated in the face of co‐infection. This project has four limiting points. (1) Mtb is a slow‐growing bacterium, which makes it difficult to obtain faster results, taking ∼60 days to obtain culture results, considering thawing of bacterial strain until definition of the intracellular bacterial load. (2) The viral stocks produced and the samples must be frozen, and they cannot be freeze‐thawed, as this can reduce the sample titer. (3) The BSL‐3 used for this experiment has a facility with essential equipment for handling the pathogens. The execution of other experiments with cell extracts from these cultures, e.g., RNA and protein, requires an additional step using reagents, such as Trizol and UV‐C, respectively, to ensure proper decontamination and no infectivity upon leaving BSL‐3. UV‐C exposure may reduce infectivity under the specific experimental conditions described in this protocol. Because UV‐C efficiency depends on factors, such as pathogen aggregation, sample depth, lamp calibration and equipment configuration, laboratories should independently validate performance according to local biosafety requirements. (4). The host cells must be suitable for continuous replication of both pathogens: Mtb and SARS‐CoV‐2. For this, the A549 cell line was selected due to the presence of cell surface receptors, which allow the entry of Mtb, in addition to expressing the angiotensin‐converting enzyme 2 (ACE2) receptor, being suitable for the entry and replication of SARS‐CoV‐2. See Table 1 for a troubleshooting guide for executing Basic Protocol.

**Table 1:** Troubleshooting Guide for Mtb and SARS‐CoV‐2 Coinfection Protocol in Human Lung Epithelial Cells

Step	Problem	Possible reason	Possible solution
6	OD between 0.85 and 0.99 nm before 28 days	High turbidity	Perform a subculture of the bacterial culture by inserting 2 ml of bacteria and 8 ml of 7H9 medium into a new tube; place in a shaker incubator at 37°C; after 3 days, check the OD
	OD >1.0 before 28 days	Bacterial death	Perform a new bacterial culture
17	Cells do not recover from freezing	Residual DMSO in cell culture medium	Always resuspend thawed cells in excess fresh maintenance medium (10 ml) to dilute the DMSO, then pellet the cells and resuspend the pellet in 10 ml fresh maintenance medium; do not plate on freezing medium and change the medium the next day
17	A549 cells did not reach 90% confluence	Bottle size	A549 cells grow in clumps and should be seeded in T25 flasks to form a compact, confluent monolayer; large flasks do not allow for optimal growth due to excess space, which can lead to cell death when trying to increase confluence to 90%
26	Very high/low cell density	Error during calculation of number of seeded cells	Check your notes and calculations and repeat the correct number
33	Empty crescent‐shaped spots in wells after staining for plaques	Drying of cells during incubation	Shake plates every 5‐10 min during incubation to prevent cells from drying out
58	Difference >5% from viral titer calculation	Pipetting	Be sure to vortex and pipette at the same frequency for all dilutions
100	After 48 hr of inoculation, there was growth of other microorganisms	Contamination	Make a control plate to check which possible reagent is contaminated and redo the experiment
			If the contaminated reagent is 7H9 medium, lysis buffer or dilution buffer, a new Mtb and infection culture should be performed
	There was no growth of Mtb after 28 days	7H10 medium supplemented with OADC at a temperature >40°C	Prepare a new 7H10 medium, ensuring that the temperature is at 40°C before adding the OADC supplement
		MOI very low	Efficient infection of Mtb in A549 cells should be MOI 10:1; make sure and redo the calculations of cell concentration per well and bacterial concentration
		Cells were not lysed	When adding a lysis buffer to the wells, pipe it vigorously and consistently; ensure that they are lysed under the microscope by observing the swollen cells
121	Presence of CPE in samples to be decontaminated	UV‐C radiation time	Use a radiometer to check how long it takes to reach the intensity and dosage required for decontamination; after determining this time, the 15 min of radiation should be counted

### Understanding Results

#### Co‐infection Mtb + SARS‐CoV‐2

After successful cultivation of Mtb and SARS‐CoV‐2 individually, we performed mono‐ and co‐infections in the host cell, and after 72 hr of completed infections, we visualized the effects on the cells via microscopy. CPE in A549 cells used in Basic Protocol will present as cell death resulting in cell rounding and detachment. CPE induced in mono‐ and co‐infections can be distinguished due to super confluence of cells and therefore comparison with uninoculated control cells plated at the same time is critical.

#### Determination of intracellular bacterial load

After mono‐infection with Mtb and co‐infection with Mtb + SARS‐CoV‐2, we expect to detect the intracellular bacterial load through CFUs to confirm the viability of the system, and to determine the rate of infection and spread between Mtb and Mtb + SARS‐CoV‐2. The amount of bacterial load may vary due to the dilutions used in the inoculation of the 7H10 agar medium. This load determination may also fail in some cases due to the preparation of the plates with 7H10 medium or due to the low number of bacteria used. To ensure the success rate of infection, we recommend using the MOI of 10 for each well of infected cells.

#### Determination of the infectious titer by SARS‐CoV‐2

In mono‐infection with SARS‐CoV‐2 and Mtb + SARS‐CoV‐2 co‐infection, we expect the presence of CPE to be observed in order to, as well as the determination of the bacterial load, to confirm the viability of the system, and verify the rate of infection and spread in SARS‐CoV‐2 and Mtb + SARS‐CoV‐2.

#### Obtaining cell extracts

We hope to obtain RNA and protein samples with a high level of purity, quality, and efficiency, providing experimentalists with materials to be used in other activities and to obtain other results, according to the objectives.

#### UV‐C decontamination

UV‐C exposure may reduce infectivity under the specific experimental conditions described in Basic Protocol. Because UV‐C efficiency depends on factors, such as pathogen aggregation, sample depth, lamp calibration and equipment configuration, laboratories should independently validate performance according to local biosafety requirements.

### Time Considerations

Stage 1: Mtb culture, takes up to 28 days after thawing. Steps 1 to 3, preparation of bacterial culture, takes 1 hr for inoculation and 4 days for incubation. Steps 4 to 6, bacterial culture maintenance, takes 1 hr and optimal growth culture takes up to 28 days.

Stage 2: Cell maintenance, takes 10 days for thawing and confluence of cells. Steps 7 to 17, thawing of cells, takes 1 hr for thawing and inoculation, and 2 to 3 days for growth and 90% confluence. Steps 18 to 28, replating of cells, takes 1 hr for cell division and 4 to 6 days for two passages.

Stage 3: Preparation of viral stocks by TCID_50_, takes 5 days total. Steps 29 to 30, plating of Vero‐CCL81 cells, takes 1 hr and an overnight incubation. Steps 31 to 36, infection of Vero‐CCL81 cells with the hCoV‐19/Brazil/PE‐FIOCRUZ‐IAM4372/2021 strain, takes 2 hr for inoculation and 72 hr for incubation. Steps 37 to 43, virus harvesting, takes 2 hr.

Stage 4: Determination of infectious titers of viral stocks, takes 5 days. Steps 44 to 45, plating of Vero CCL81 cells, takes 1 hr for infection and overnight incubation. Steps 46 to 53, TCID_50_ assay, takes 2.5 hr for inoculation and 72 hr for incubation. Steps 54 to 58, reading of plates by light microscopy and calculation of viral titer, takes 2 hr.

Stage 5: Co‐infection of A549 cells, takes 5 days total. Steps 59 to 60, plating of A549 cells, takes 1 hr for plating and an overnight incubation. Steps 61 to 78, preparation of bacterial inoculum and infection of A549 cells, takes 4 hr. Steps 79 to 86, preparation of viral inoculum, infection, and co‐infection of A549 cells, takes 3 hr for infection and 72 hr for incubation. Steps 87 to 92, harvest of RNA and proteins, takes 4 hr.

Stage 6: Determination of intracellular bacterial load, takes 28 days total. Steps 93 to 100, cell lysis, dilutions, and inoculation of plates 7H10, takes 3 hr for preparation of viability and 28 days for incubation.

Stage 7: SARS‐CoV‐2 viability test by TCID50, takes 5 days total. Step 101, plating of Vero CCL‐81 cells, takes 1 hr for plating and an overnight incubation. Steps 102 to 107, rack preparation and inoculation with viral harvests (mono‐ and co‐infected) from the culture, takes 1 hr for rack preparation, 1.5 hr for infection, and 72 hr for incubation. Step 108, reading of plates by light microscopy and calculation of viral titer, takes 3 hr.

Stage 8: UV‐C decontamination, takes 5 days total. Steps 109 to 110, plating of Vero CCL‐81 cells, takes 1 hr for plating and an overnight incubation. Steps 111 to 120, preparation of supernatant aliquots, takes 2 hr for decontamination and 72 hr for incubation. Step 121, reading of plates by light microscopy, takes 1 hr.

### Author Contributions

**Thays Maria Costa de Lucena**: Conceptualization; formal analysis; methodology; writing—original draft. **Débora Elienai de Oliveira Miranda**: Methodology. **Juliana Vieira de Barros Arcoverde**: Methodology. **Mariana Souza Bezerra Cavalcanti**: Methodology. **Willyenne Marilia Dantas**: Methodology. **Lindomar José Pena**: Methodology; validation; writing—review and editing. **Michelle Christiane da Silva Rabello**: Methodology; validation; writing—review and editing. **Jaqueline de Azevedo Silva**: Conceptualization, supervision, validation, writing—original draft; writing—review and editing.

### Conflict of Interest

The authors declare that they have no known competing financial interests or personal relationships that could have appeared to influence the work reported in this article.

## Data Availability

The data that support the findings of this study are available from the corresponding author upon reasonable request. This manuscript is hosted in the bioRxiv repository at: https://doi.org/10.1101/2024.08.14.607914.
